# Identification of a broad lipid repertoire associated to the endothelial cell protein C receptor (EPCR)

**DOI:** 10.1038/s41598-022-18844-y

**Published:** 2022-09-06

**Authors:** Elena Erausquin, María Morán-Garrido, Jorge Sáiz, Coral Barbas, Gilda Dichiara-Rodríguez, Alejandro Urdiciain, Jacinto López-Sagaseta

**Affiliations:** 1 Unit of Protein Crystallography and Structural Immunology, Navarrabiomed, 31008 Pamplona, Navarra Spain; 2 Public University of Navarra (UPNA), 31008 Pamplona, Navarra Spain; 3 Navarra University Hospital, 31008 Pamplona, Navarra Spain; 4 Centre of Metabolomics and Bioanalysis (CEMBIO), Department of Chemistry and Biochemistry, School of Pharmacy, Universidad San Pablo-CEU, CEU Universities, Urbanización Montepríncipe, 28660 Boadilla del Monte, Spain

**Keywords:** Lipidomics, Structural biology

## Abstract

Evidence is mounting that the nature of the lipid bound to the endothelial cell protein C receptor (EPCR) has an impact on its biological roles, as observed in anticoagulation and more recently, in autoimmune disease. Phosphatidylethanolamine and phosphatidylcholine species dominate the EPCR lipid cargo, yet, the extent of diversity in the EPCR-associated lipid repertoire is still unknown and remains to be uncovered. We undertook mass spectrometry analyses to decipher the EPCR lipidome, and identified species not yet described as EPCR ligands, such as phosphatidylinositols and phosphatidylserines. Remarkably, we found further, more structurally divergent lipids classes, represented by ceramides and sphingomyelins, both in less abundant quantities. In support of our mass spectrometry results and previous studies, high-resolution crystal structures of EPCR in three different space groups point to a prevalent diacyl phospholipid moiety in EPCR’s pocket but a mobile and ambiguous lipid polar head group. In sum, these studies indicate that EPCR can associate with varied lipid classes, which might impact its properties in anticoagulation and the onset of autoimmune disease.

## Introduction

EPCR is a transmembrane MHC class I-like glycoprotein primarily abundant in the vasculature of large vessels^1^, but present in numerous cell types. Apart from the endothelial cell lining, EPCR is found in eosinophils^2^, neutrophils^3^, monocytes^4^, keratynocytes^5^, neurons^6^, bone marrow and hematopoietic stem cells^7,8^, cardiomyocytes^9^, vascular smooth muscle cells^10^ or placental trophoblasts^11^. Since its identification, EPCR has been mainly known for its role in accelerating the generation of the anticoagulant activated protein C (APC) and favoring its cytoprotective properties^12–16^. More recent studies have linked EPCR to other scenarios, such as severe malaria, as EPCR mediates gripping to membrane proteins in infected erythrocytes^17^. In a more immunological setting, two studies have described subsets of γδ T cells that target infected cells via EPCR. The first study reports cytomegalovirus-infected endothelial cells and epithelial tumors as γδ T cell targets^18^, while a second and more recent study defines a role for EPCR in the recognition of mast cells upon infection with dengue virus^19^. Further, EPCR has been associated with autoimmune disease and related coagulopathies, as proved by several groups reporting the presence of anti-EPCR autoantibodies in antiphospholipid syndrome^20,21^ and ulcerative colitis^22^.

Structurally, EPCR shares a notable degree of homology with the CD1 family of receptors, including the presence of a central and extended hydrophobic cavity suitable for lipid antigens, flanked by two alpha helices lying on top of a beta sheet. The high-resolution structures reported by Oganessyan and coworkers^23^ provided solid evidence for the presence of a phosphatidylethanolamine (PE) molecule bound in the EPCR lipid cleft. More specifically, it is shown that the two acyl chains are buried inside the hydrophobic pocket, while the polar head group is outwardly positioned, and therefore accessible for potential binders. Later studies showed that phosphatidylcholine is abundant in EPCR, and that certain lysophospholipids can also bind to EPCR and modulate its anticoagulant and cytoprotective properties^24^, which gave rise to the concept of *EPCR encryption*^25^. Indeed, lysolipid molecules show an affinity for EPCR greater than that of diacyl-PC, with K_D_ values ranging 1 to 8 µM for monoacyl lysophosphatidylcholine species, respectively, while PC’s K_D_, 108 µM, mirrored a lower affinity^24^. These results suggest that the fitting of diacyl-PC species within the EPCR groove has a greater structural restriction. The affinity of lysolipids differ only slightly among them as a function of the number of carbons in the acyl chain as well as the presence of insaturations: 16-carbon lysoPC with 1 saturation represented the strongest binder.

The closely related lipid antigen-presenting CD1 molecules are targeted by T cell receptors (TCRs) in a lipid antigen-dependent manner. Antigens presented by CD1d are in their vast majority glycolipids and phospholipids, both natural or synthetic, self or foreign^26–28^. Importantly, membrane-anchored EPCR can internalize, enter the recycling pathway and return to cellular surface^29^. This well-known process in antigen-presenting major histocompatibility complex (MHC) and CD1 molecules enables renewal of peptide (MHC) and lipid (CD1) loads prior to replacement in the cell membrane, where the newly filled antigens are molecularly accessible for immune receptors^30^. It is tempting to speculate, therefore, about the existence of immune receptors directed against EPCR in a lipid-restricted manner. A recent study reports the recruitment of autoantibodies in antiphospholipid syndrome in an EPCR and lipid dependent manner^21^. Müller-Calleja and coworkers inform that lysobisphosphatidic acid, present in endosomal compartments, loads into EPCR and triggers recruitment of antiphospholipid autoantibodies, which represents a novel mechanism in the etiology of this autoimmune disease.

In sum, there are growing evidences that the nature of the lipid bound to EPCR could have a relevant impact on disorders related with coagulation and autoimmune disease. To contribute to the elucidation of the types of lipids that can associate with EPCR, we have carried out mass spectrometry and structural studies, and present here the EPCR lipidome, which includes novel lipid classes not yet linked to this receptor.

## Results

### The EPCR lipidome

**Figure 1:**
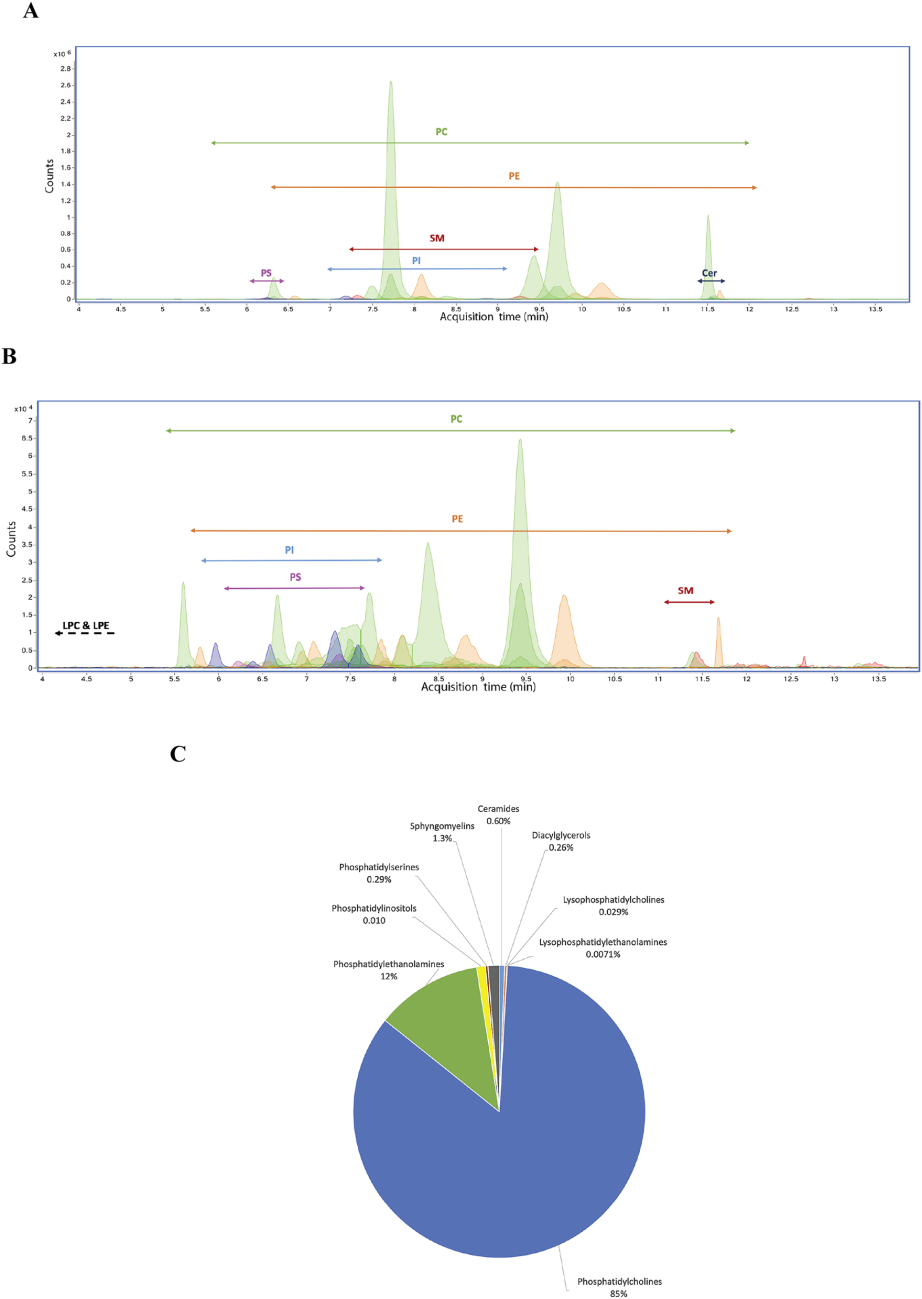
Extracted ion chromatogram of relevant lipids found in the native EPCR. Retention times associated to each lipid class identified in the sEPCR organic extract. The arrows indicate the chromatographic region where each lipid class elutes. The figure is divided showing lipids with major (**A**) and minor (**B**) abundances in the EPCR pool of extracted lipids. The experimental conditions are described in section Materials and Methods in the supplementary data. (**C**) distribution of the different EPCR lipid classes.

The human extracellular region of EPCR (hereafter referred as EPCR) was produced in sf9 insect cells and purified to homogenity (Supplementary Fig. 1). Subsequently, the lipid content was isolated with a standard procedure based on organic solvents and the lipid fractions were dessicated under a stream of nitrogen prior to mass spectrometry analyses. Liquid chromatography–mass spectrometry (LC–MS) analyses yield a total of 41 different lipids identified (Table S1 and Fig. 1), of which 38 and 32 were determined in positive and negative ionization mode, respectively. Thirty-one of these lipids could be detected in both ionization modes providing a greater confidence in their identification.

Up to four independent samples were prepared and analyzed and the lipids present in at least three of those samples are described here, along with their relative abundances (Fig. 1). The main lipid category found in EPCR was the glycerophospholipid (GPL) class, counting up to 98% of the total lipid population. Within this class, phosphatidylcholines (PC) constitute the most abundant species (Fig. 1A and C), making up to 85% of the total lipids. Phosphatidylethanolamines (PE) (12%), phosphatidylinositols (PI) (0.96%), phosphatidylserines (PS) (0.29%) and lyso forms containing just one acyl chain, such as lysophosphatidylcholine (LPC) (0.029%) and lysophosphatidylethanolamine (LPE) (0.0071%), were also found in smaller amounts. Ceramides (Cer), sphingomyelins (SM) and diacylglycerols (DG) were found as well in very small proportions (0.60%, 1.3% and 0.26%, respectively (Figs. 1B and C). In a control sample containing the insect cell culture growing medium we could only detect fatty acids.

**Figure 2:**
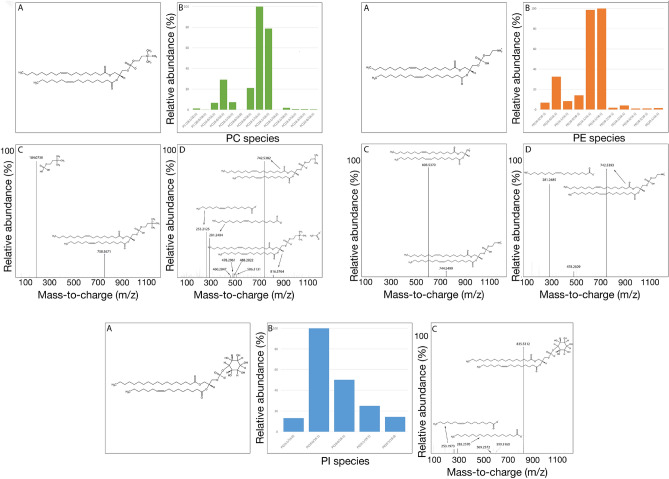
Major phospholipid species extracted from EPCR. A schematic structure for the most abundant species and their distribution is shown for PC (**A**), PE (**B**) and PI (**C**) species, as determined by LC–MS analysis. Fragmentation patterns in positive (PC and PE) and negative (PC, PE and PI) ionization modes are included.

The identification of each lipid was based on their m/z, MS/MS spectra (Fig. 2) adduct formation distribution, and collisional cross section (CCS) values obtained from ion mobility. The CCS values for selected adducts of these lipids were obtained (Table S1), and confirmed in the CCSBase (https://ccsbase.net). The average error associated to each experimental CCS determination was 0.37%, which is in agreement with the error associated to single-field CCS determination^31^. Fitting of the data was excellent (Figure S2), supporting the ion mobility data for lipid identification.

**Figure 3:**
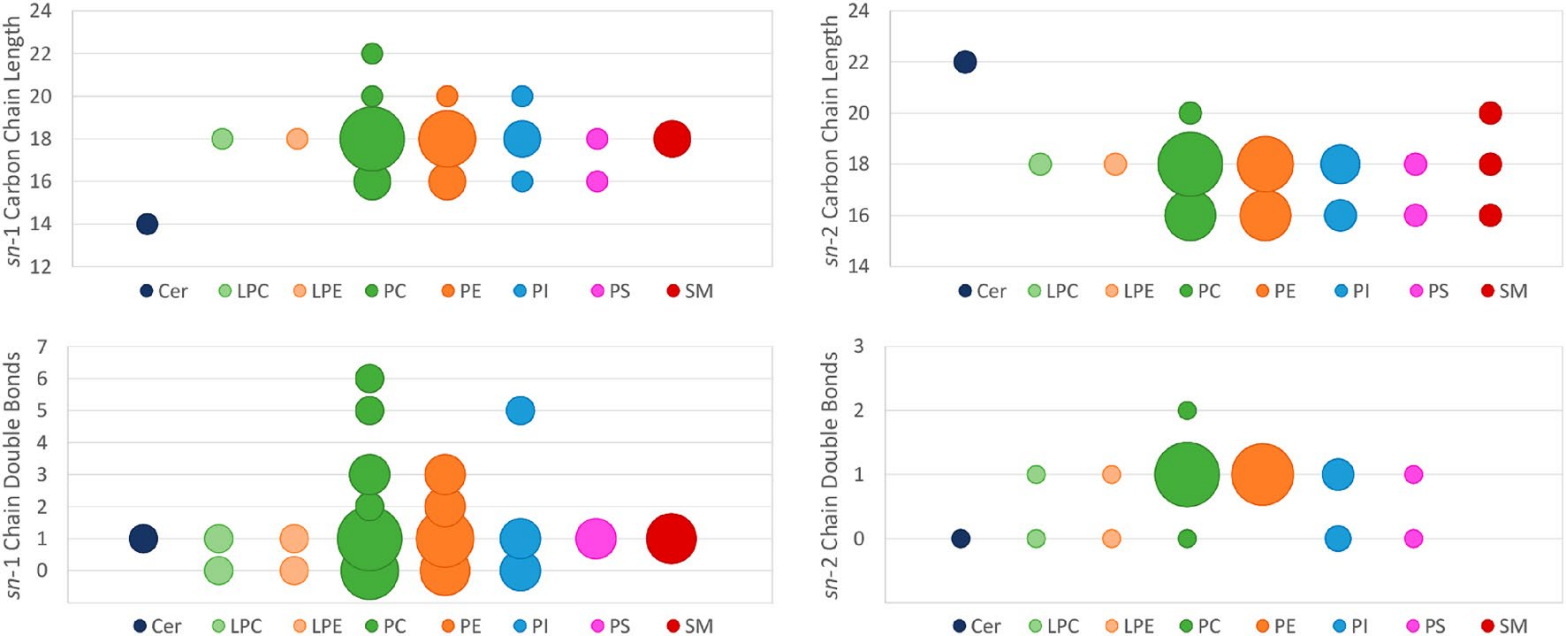
Acyl chain lenghts and unsaturation degree in each lipid class. Y-axis represents the characteristic analyzed and x-axis contains each lipid class with each corresponding color. Bubble sizes are proportional to the number of lipids at each data point. (**A**) Analysis of the sn-1 carbon chain length in each lipid class. The most common length was of 18 carbon for most lipid classes. (**B**) Analysis of the sn-2 carbon chain length in each lipid class. The most common lengths were of 18 and 16 carbon for most lipid classes. (**C**) Analysis of the sn-1 chain double bonds, regardless of the chain length. Most lipid classes had either 0 or 1 double bond in sn-1. (**D**) Analysis of the sn-2 chain double bonds, regardless of the chain length. Most lipid classes had 1 double bond in sn-2.

Analyzing acyl chain compositions, several chain lengths and double bond distributions could be found, especially in the major lipid classes. Acyl chains of 16 and 18 carbons were the most frequent in both sn-1 and sn-2 positions (Fig. 3), being present in basically all lipid classes. Longer chains could be found, mainly in the sn-1 position and never exceeding 22 carbon atoms. Interestingly, as opposed to Cys13 and Leu161 in CD1d, which allows arrangement of sn-1 acyl within the A´ pocket, EPCR contains bulkier methionine and phenylalanine residues. This suggests a more severe restriction for long acyl groups in EPCR (Figure S3). Regarding unsaturations, we found a major population defined by 1 unsaturation in the sn-1 acyl chain. The heterogeneity encompasses lipids from 0 to 6 unsaturations in sn-1. As for sn-2, this heterogeneity is reduced, and we only detected species with 0 to 2 unsaturations, being 1 the most representative. Altogether, the length and number of unsaturations detected mirrors a lipid cavity suitable for the binding of diverse lipids, where 18-carbon long PC and PE species with a low number of unsaturations are the dominant species (Fig. 3).

Consequently, the major lipid bound to EPCR was PC 18:1/16:1, closely followed by PC 18:1/18:1 and PC 16:0/18:1. Distribution of PE was near identical to that of PC. Although much less abundant than PC; PE 18:1/18:1, PE 18:1/16:1 and PE 16:0/18:1 were the three major PE species. In conclusion, our analyses reveal a heterogeneous array of lipids with novel species isolated from EPCR.

### X-ray diffraction studies suggest phospholipid heterogeneity

**Figure 4:**
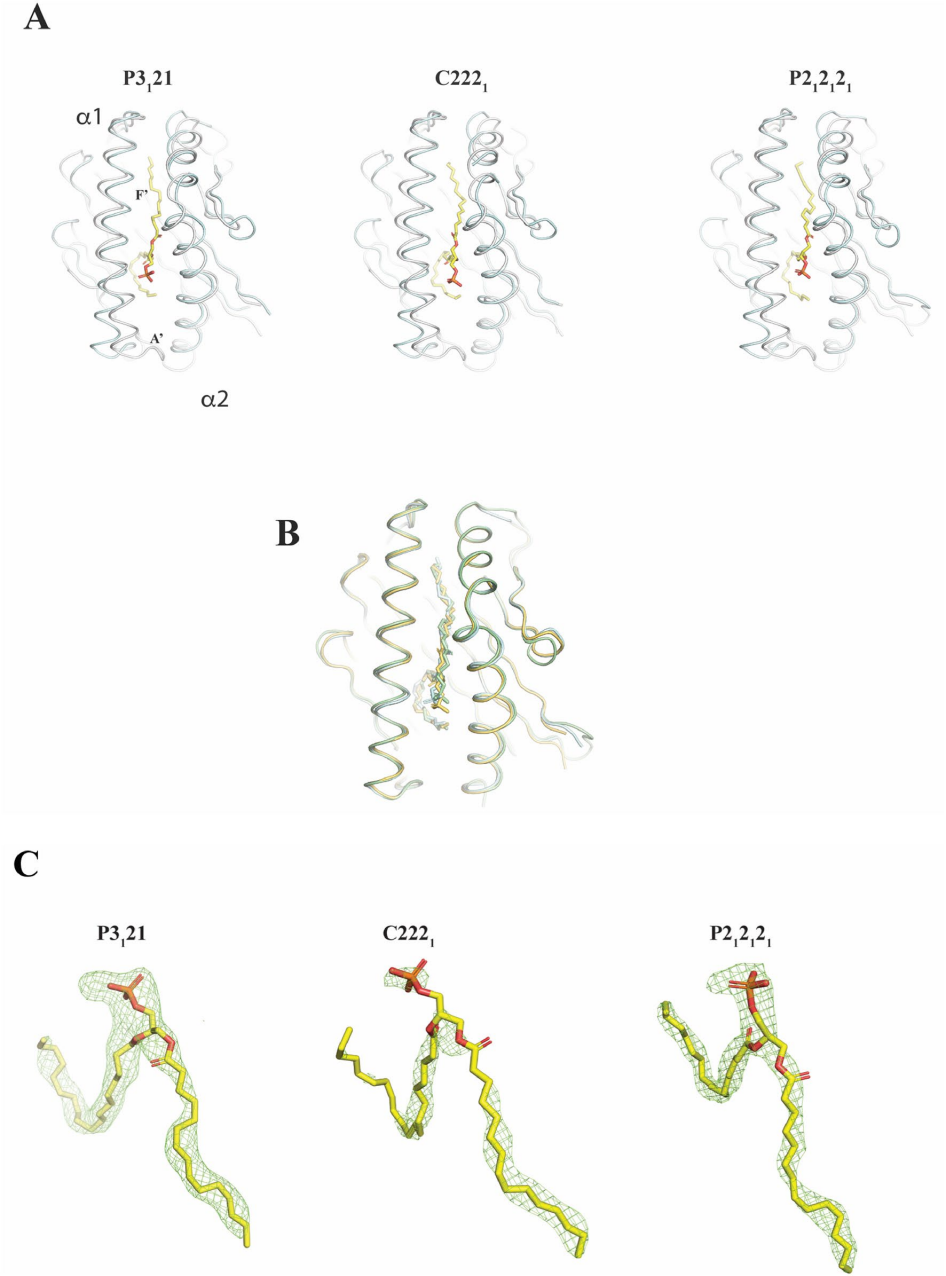
Superposition of EPCR and CD1d crystal structures. (**A**) Ribbon diagram showing a top view of human CD1d structure (light gray), extracted from coordinates in Protein Data Bank deposition 4EN3, superposed with EPCR structures (palecyan colored) crystallized in space groups P3_1_21 (left), C222_1_ (middle) and P2_1_2_1_2_1_ (right). A phospholipid molecule is shown in sticks mode, where the outer most polar group is not shown. (**B**) Superposition of EPCR molecules crystallized in the three different groups shown in (**A**). Colors are palegreen, yelloworange and palecyan for molecules in P3_1_21, C222_1_ and P2_1_2_1_2_1_ space groups, respectively. (**C**) Heterogeneity in the EPCR lipid binding cleft. Fo-Fc difference omit maps (shown as green colored meshes) were generated through refinement using full EPCR, ligands and water coordinates and omitting the lipid molecule. Given the lack of electron density signal, the phospholipid molecule does not include the group (-choline, -ethanolamine, -serine or -inositol) linked to the phosphate. All figures were prepared with PyMOL version 2.5.2.

We undertook X-ray diffraction studies that allowed analysis of the cavity content using a different approach. For this, we performed a wide crystallization screen and obtained EPCR crystals in varied conditions, which ultimately led to solution of the structure in three different space groups. The resolution in all cases was set to 1.95 Å (Table 1), with data completeness above 99% and half-split Pearson correlation coefficients (CC_1/2_) values greater than 0.5 in the high-resolution shells. Out of the three different space groups, P3_1_21 recapitulates a previous structure crystallized in the same space group^23^, with 1 molecule of EPCR in the asymmetric unit. Crystals grown in other conditions yielded EPCR structures in two novel space groups, C222_1_ and P2_1_2_1_2_1_, containing 2 and 4 molecules of EPCR per asymmetric unit, respectively. Thus, crystallization in different space groups enabled a broader assessment of the EPCR’s lipid content. With the exception of disordered regions, the electron density maps accurately traced the EPCR overall architecture, backbone and side chains (Figs. 4A and B). Structural alignment against EPCR in space group P3_1_21 yielded root mean square deviations of 0.459 and 0.400 for EPCR molecules in space groups C222_1_ and P2_1_2_1_2_1_ (Fig. 4B)_._ EPCR shows in all cases a conserved CD1-like α1-α2 scaffold depicting two alpha helices sitting over a beta sheet and creating a hydrophobic tunnel-like cleft for lipid binding. As for CD1 molecules, two pockets, referred as A' and F', are found in EPCR’s groove and provide adequate chemistry for accommodation of alkyl-based ligands such as diacyl lipids. We observed, however, non-homogeneous signals inside the lipid cavities. EPCR in P3_1_21 and P2_1_2_1_2_1_ space groups present strong and continuous Fobs-Fcalc difference maps that suggest a prevalent diacyl molecule but a more ambiguous head group moiety (Fig. 4C). Fitting of a diacyl phospholipid scaffold (lacking the outermost head group) satisfied the overall shape of the map, albeit with some degree of uncertainty for the orientation of the carbonyl groups. Despite the high resolution, we observed a less accurate electron density map in one of the two EPCR molecules in space group C222_1_, where the signal appears disconnected (Fig. 4C) and fitting of a diacyl phospholipid results less satisfactory.

**Table 1:** X-ray data collection, processing, and refinement statistics.

PDB code	7OKS	7OKT	7OKU	7OKV
Wavelength (Å)	0.97926	0.97926	0.97926	0.97934
Resolution range	39.22–1.95 (2.02–1.95)	42.94–1.95 (2.02–1.95)	38.09–1.95 (2.02–1.95)	35.53–1.85 (1.92–1.85)
Space group (Å)	P2_1_2_1_2_1_	C222_1_	P3_1_21	P3_1_21
Unit cell a, b, c (Å)	78.43 94.019 124.595	75.295 91.602 123.36	70.619 70.619 97.367	71.051 71.051 103.153
Unit cell α, β, γ (°)	90 90 90	90 90 90	90 90 120	90 90 120
Total reflections	439,867 (45,612)	203,398 (19,437)	203,998 (19,902)	212,872 (21,706)
Unique reflections	67,764 (6692)	31,444 (3080)	21,014 (2065)	26,218 (2571)
Multiplicity	6.5 (6.8)	6.5 (6.3)	9.7 (9.6)	8.1 (8.4)
Completeness (%)	99.65 (99.69)	99.88 (99.94)	99.96 (99.95)	99.51 (98.80)
Mean I/sigma(I)	10.88 (1.71)	12.97 (0.98)	15.05 (0.90)	13.90 (1.31)
Wilson B-factor	36.27	45.37	52.51	37.80
R-merge	0.0867 (1.072)	0.0591 (1.498)	0.0640 (2.09)	0.09006 (2.223)
R-meas	0.0945 (1.162)	0.0644 (1.634)	0.0677 (2.208)	0.09642 (2.366)
R-pim	0.0371 (0.4438)	0.0253 (0.6478)	0.0218 (0.7081)	0.03399 (0.804)
CC1/2	0.998 (0.883)	0.999 (0.562)	0.998 (0.58)	0.998 (0.624)
Reflections used in refinement	67,597 (6676)	31,413 (3079)	21,009 (2064)	26,185 (2563)
R-work	0.1990 (0.3373)	0.2206 (0.3597)	0.2045 (0.3130)	0.2131 (0.3545)
R-free	0.2393 (0.3733)	0.2565 (0.3698)	0.2367 (0.2923)	0.2310 (0.3851)
Number of non-hydrogen atoms	6020	2710	1456	1458
Macromolecules	5350	2492	1330	1324
Ligands	475	192	119	78
Solvent	195	26	7	56
RMS(bonds)	0.010	0.010	0.012	0.014
RMS(angles)	1.09	1.04	1.26	1.48
Ramachandran favored (%)	97.25	97.12	95.29	96.32

Values in parentheses are for highest resolution shell.

Thus, in agreement with the MS results, the diffraction studies of our EPCR crystals suggest that varied phospholipids fill the EPCR’s hydrophobic pocket.

### The polar regions of the lipids bound to EPCR are not structurally locked

**Figure 5:**
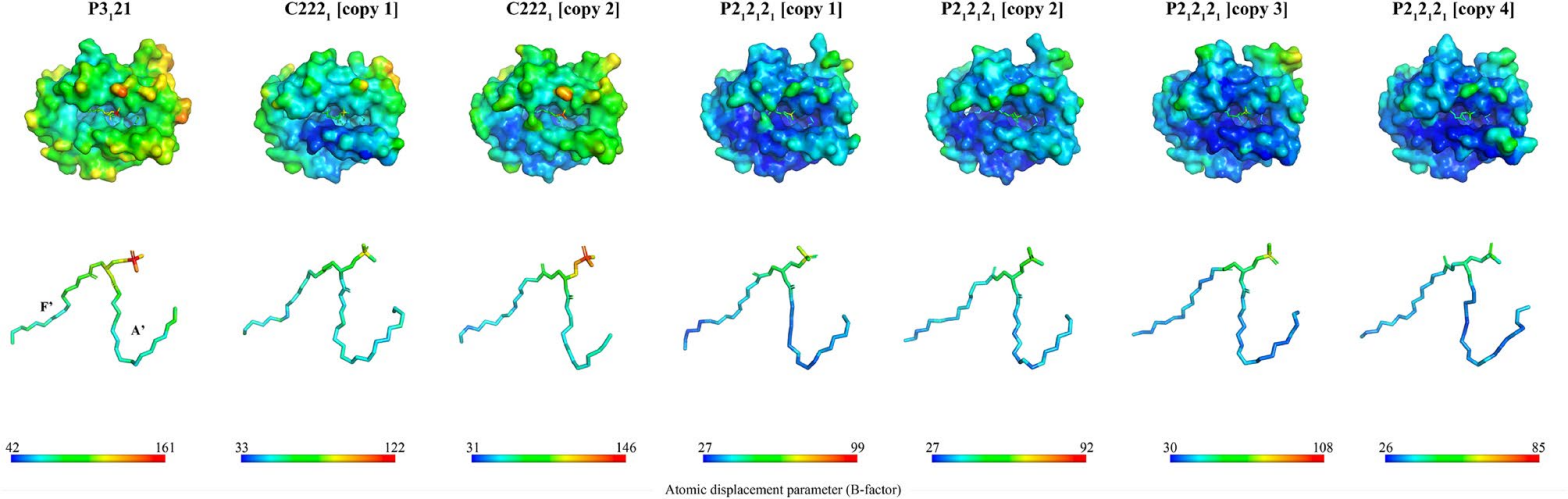
Mapping of the static disorder in the EPCR-lipid complexes. All EPCR molecules are shown in surface format, and colored according to their ADP (B-factor) value, which is a measure of the atomic disorder from a mean position. The B-factor scale is displayed as a colored scale bar showing the minimum and maximum values for each structure. To ease visualization of the B-factor gradient, the lipid molecule for each structure is extracted in the middle panel. All structural figures and scale bars were prepared with PyMOL version 2.5.2.

The lack of a solid electron density in the lipid cavity, as seen in some of the crystal structures, could be an indication not only of lipid heterogeneity, but unstable lipid binding. In order to assess the later, we mapped the atomic displacement parameters (ADPs, also known as B-factors) in all space groups in which EPCR was crystallized (Fig. 5). Persistent lower B-factor values were observed for the buried fatty acids, shielded inside the A’ and F’ hydrophobic pockets, and suggesting locked, stable binding. On the contrary, the B-factor values are higher for the atoms in the polar regions, which locate near the EPCR groove opening, thus indicating less stable or disordered binding. The P3_1_21 space group yield the highest B-factors for all atoms, while those EPCR-lipid complexes present in the P2_1_2_1_2_1_ crystal structure showed the most rigid atom positions, as suggested by the lower B-factor values. These results suggest that EPCR lipids are tightly bound through the acyl chains but a less stable interaction is achieved around the polar region.

### The morphology of EPCR’s lipid cavity is malleable

**Figure 6:**
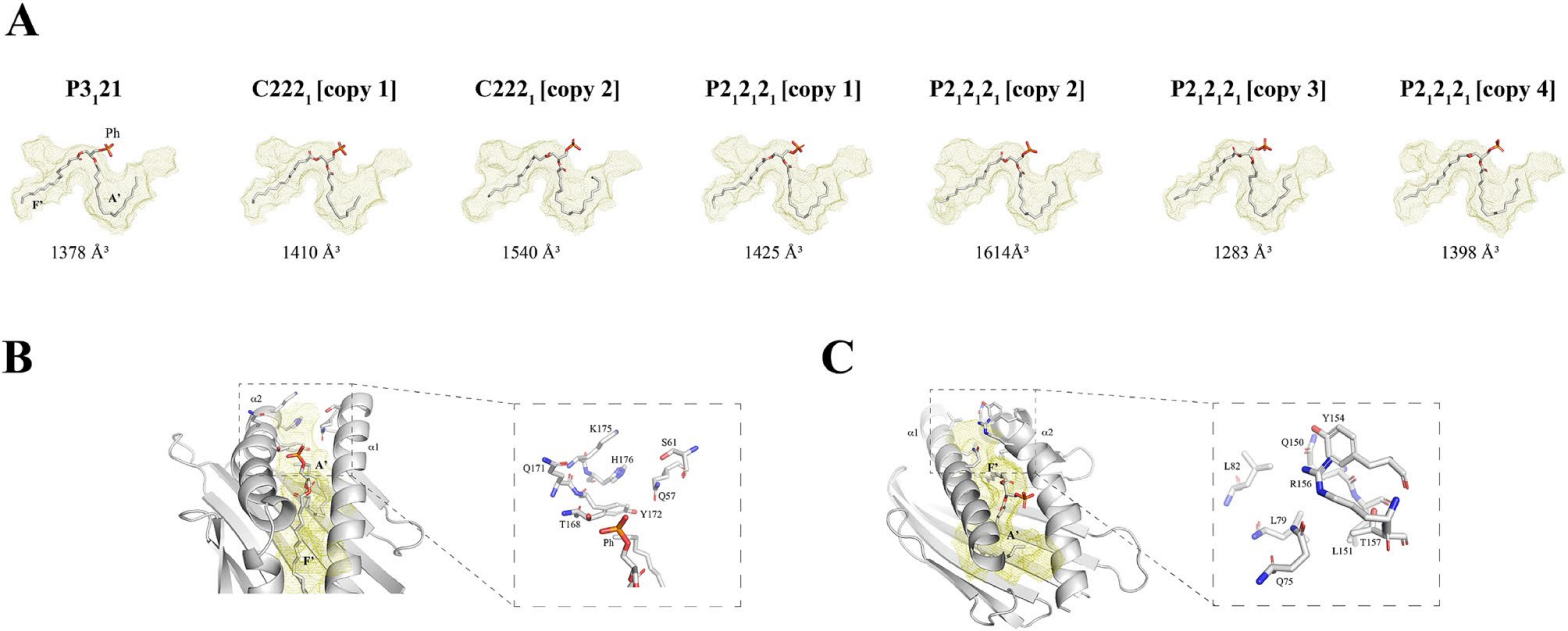
Morphology of the EPCR lipid-binding cavity. (**A**) The space that depicts the lipid pocket in each EPCR structure is displayed as a paleyellow mesh, with the phospholipid (Ph) molecule inside, as it is present in the crystal structure. The A’ and F’ pockets are indicated in the P3_1_21 structure. Pockets were determined with DoGSiteScorer^55^ (**B**) Visualization of the additional pocket detected in EPCR near A’. Shown is the structure corresponding to space group P2_1_2_1_2_1_, copy 4, in grey cartoon representation. The residues that surround each pocket are highlighted in stick format, and additionally, in a zoomed-in side image. (**C**) As in (**B**), the smaller pocket found beyond F’ is indicated and further detailed in a zoomed image. All figures were prepared with PyMOL version 2.5.2.

Lastly, we looked at the geometric properties of the binding pockets in all space groups (Fig. 6A) and compared their shapes and volumes. Amongst all EPCR molecules in the three space groups obtained, the dimensions of the pockets range from ~ 1280 to ~ 1615 Å^3^, where both values were found in two of the four EPCR molecules present in the P2_1_2_1_2_1_ space group asymmetric unit. The only molecule found in P3_1_21 yield a ~ 1375 Å^3^ cavity while the two molecules in the asymmetric unit of C222_1_ have pockets of 1410 and 1540 Å^3^. As a reference, the EPCR molecule previously crystallized^23^ in space group P3_1_21 was calculated to have a ~ 1550 Å^3^ cavity. Although with a tendency to be smaller, these values fall within the range found for the pockets of the CD1 family of lipid-binding receptors^32^.

Two main pockets of hydrophobic nature can be discriminated as part of the morphology of the lipid cavity, denoted as A’ and F’, where the acyl chains are allocated. Although not hydrophobic in their chemistry, additional empty spaces are observed in two additional smaller pockets beyond A’ and F’ (Fig. 6B,C). While the extra space near F’ is present in all EPCR structures presented herein, the additional pocket close to A’ is only shown in some molecules.

In conclusion, our crystallization studies provide an alternative view that, like our mass spectrometry results, indicate that EPCR presents a plastic cavity that associates with a heterogeneous and mobile population of phospholipids.

## Discussion

The presence of a lipid bound to EPCR is key for its biological functions. Oganessyan and colleagues provided solid evidence, on one hand, for the presence of PE bound to EPCR through a high-resolution crystal structure, and on the other hand, for the relevance of the bound lipid in EPCR’s functions, as removal of the lipid fraction led to loss of its ability to bind APC^23^. A later work expanded these results, showing first, the predominant presence of PC in the lipid cargo of EPCR, second, the ability for lipid exchange and third, the lipid-dependent APC activation and antiapoptotic properties of EPCR^24^. Of note, EPCR has also been associated with autoimmune disease. In particular, with coagulopathies linked to the antiphospholipid syndrome^20,21^, and on a different ground, with ulcerative colitis^22^. These studies indicate that EPCR is an autoantigen capable of recruiting autoantibodies, which could ultimately contribute to the onset and course of inflammatory response in autoimmune disease. Moreover, a recent study reports that a well-defined lipid, lysobisphosphatidic acid, present in the endosomal compartment of monocytes, loads into EPCR and triggers binding of antiphospholipid antibodies on the cell surface^21^. Given these precedents, a deep understanding on the classes of lipids that EPCR can bind is therefore of interest. We previously investigated the lipid content via thin layer chromatography^24^. This technique, combined with staining of organic molecules with phosphomolybdic acid allowed us detect the most populated lipids in EPCR samples: PC and PE. Phosphomolybdic acid is a widely used chemical for detection and visualization of a large variety of organic compounds, including phospholipids. However, the identification of other compounds present in less abundant quantities might be compromised. To deepen into the lipids that conform the EPCR lipidome and which could be in lower quantities, we undertook a mass spectrometry approach. In agreement with previous results, the most abundant lipids found where PC and PE. These are represented by species containing varied acyl chain lengths and unsaturations. The presence of a phospholipid molecule is consistent with previous results^23^ via X-ray crystallography, where the structural analyses provide documented evidence for a phospholipid molecule bound to EPCR. The exact nature of this molecule becomes evident in the structure of EPCR complexed with the Gla domain of protein C (PDB accession code 1LQV). On the other hand, the structure of EPCR without the PC Gla domain (PDB accession code 1L8J) shows ambiguous electron density at the lipid polar region, whereby the head group of the phospholipid could not be accurately determined. This suggests that the EPCR protein co-crystallized with a non-homogeneous phospholipid molecule bound in the groove. Later studies confirmed the presence of PE, but also PC, in the organic fraction released from EPCR^24^, providing the first evidences of EPCR lipid heterogeneity.

Remarkably, apart from the major phospholipid content, we also identified other lipids not previously associated with EPCR. These are phosphatidylserine (PS), phosphatidylinositol (PI) and, surprisingly, sphingomyelin and ceramides.

PS is found primarily in human brain tissues and, importantly, in the inner leaflet of cell membranes^33^. Exposure of PS upon cell injury provides and anchoring platform for various coagulation factors, a mechanism widely accepted to favor a procoagulant setting. Consequently, binding of PS to EPCR on the endothelial lining might associate with a novel mechanism linking PS with a prothrombotic setting.

As for PI, it can represent up to 10% of the phospholipid moiety in brain tissue, although it is less abundant in the membranes of eukaryotic cells^34^. Contrary to other phospholipids, PI has a unique structural motif, as it carries a myoinositol sugar group covalently linked to the lipid head. Myoinositol is a cyclic carbohydrate that differs from other phospholipids head groups in both the volume and number of functional (-OH) groups. Like myoinositol, other cyclic carbohydrates in the form of galactose and glucose are often linked to reactive CD1d-restricted NKT-cells, which can recognize and respond to lipids carrying a sugar moiety^26^. CD1d also binds PI and has been proposed to trigger self-reactivity^35^. In an analogous manner, it is tempting to speculate that this structural motif could trigger a lipid-restricted recognition of EPCR by proteins of the immune system, such as TCRs or antibodies.

EPCR properties are lipid-dependent. For instance, as described above, the presence of bound diacyl phosphoethanolamine or phosphatidylcholine is key for engagement of protein C/APC with EPCR. This is elegantly demonstrated by Oganessyan and coworkers with the 1.60 Å crystal structure of the Gla domain of protein C targeting EPCR^23^. The electron density of this complex shows excellent and unambiguous signal for PE, which defines with high accuracy the polar head group and the length of the acyl chains. In this structure (PDB 1LQV), it can be observed that Arg156 adopts a specific and rigid conformation through interactions with PE phosphate group. This interaction frees up space for the creation of a hydrophobic pocket that enables fitting of the protein C Gla Phe4 residue and consequently, docking of the protein C on EPCR (Supplementary Fig. 4). In the delipidated EPCR structure, the electron density for the Arg156 side chain is absent, which suggests high flexibility (elevated B-factors) and conformational disorder. Thus, the absence of a locked phosphate lipid head favors a loose Arg156 conformation that might likely hinder formation of the Phe4 pocket and proper interaction with protein C, as previously reported for delipidated EPCR^23,24^.

These observations that EPCR binders, such as protein C/APC, may bind preferentially to certain EPCR-lipid complexes. Moreover, and in line with the lipid dependency of EPCR, surface plasmon resonance studies showed that loading of delipidated EPCR, whose interaction with protein C/APC is compromised, with PC restored its binding ability^24^. Up to date, several structural and binding studies have documented the binding of EPCR binders near the lipid pocket. These ligands are protein C and factor VII in both their zymogen and activated forms^36–38^ and the CIDRα domains^39^ found in the *Plasmodium falciparum* erythrocyte membrane protein-1 (PfEMP1) family of proteins. A recent study also suggests engagement of EPCR by antibodies on or near the lipid binding site^21^.

Although the binding cleft in EPCR is slightly wider than that of CD1d, both proteins present cavities that are hydrophobic in nature, and this chemical environment favors interactions with non-polar lipid tails in the A’ and F’ pockets, as defined for CD1d. We have seen that the most abundant species bound to EPCR carry lipid tails that are no longer than 22 carbons. Unlike CD1d that can host lipids as long as 26 carbons in the A' pocket, as it is the case of alpha-galactosylceramide^40,41^, for instance, we did not find lipids with tails larger than 22 carbons in the lipid load of EPCR. A reasonable basis for this discrepancy can be found in the structural differences in the A' pocket of EPCR with respect to that of CD1d. The presence of bulkier Met13 and Phe164 in EPCR's groove near the edge of the phospholipid tail in the A' pocket, as opposed to Cys and Leu in CD1d, may confer a more severe restriction. In this line, the frequency of 22 and 24-carbon long lipids in CD1d is higher^42^. However, and similar to CD1d, we found that acyl chains of 18 carbons in both the sn-1 and sn-2 tails dominate the EPCR lipid sample. Although in a lower extent, we could also detect species with 16-carbon long acyl chains in the family of lysophospholipids, diacyl-phospholipids and sphingomyelins. Other minor species present shorter chains, like 14-carbon long ceramides, or longer, as detected for some ceramides (22 carbon in sn-2). Interestingly, in the lysophospholipid group, we could only detect 18-carbon long species in either sn-1 or sn-2. Nevertheless, as for CD1d^42^, phospholipid species represent a major component of the EPCR lipid signature.

We also analyzed the extent of unsaturations. Most sn-1 chains had either 0 or 1 double bond, but higher degrees of unsaturations were detected, in some species, presenting up to 6 double bonds. Interestingly, unlike CD1d^42^, that yields lipids with up to 6 unsaturations in sn-2, EPCR shows a much lower complexity, whereby 1 double bond is the most prevalent unsaturation and lipids with more than 2 unsaturations are not detected. The fact that the source (cell lines) were these two proteins were produced are different could contribute to these discrepancies. CD1d was expressed in a human B cell line, while EPCR has produced in *Spodoptera frugiperda* sf9 cells.

Of particular interest is the identification of ceramides and sphingomyelin. These are structurally related with other sphingolipids such as hexosylceramides or lactosylceramides, among other types of lipids. Despite the presence of this class of molecules, we did not find glycolipids with sugar groups conjugated to ceramide backbones. The lack of such species could denote a structural restriction in EPCR caused by bulky sugar groups incompatible with fitting into EPCR’s binding pocket. However, the detection of inositol-containing phospholipids in the EPCR lipidome does support this possibility. In this line, in a previous report, Marheineke K. et al.^43^ analyzed the lipid content in sf9 cells, the cell type where we produce our recombinant EPCR, and could not detect glycolipid species. Thus, an alternative reason for the absence of glycolipids in the EPCR lipidome is the lack of a source for this specific lipid class. Likewise, we did not detect phosphatidic acid in our samples, but the presence of this molecule was not reported either in the sf9-derived lipid pool.

To explore the content of the cavity through a different approach, we carried out an extensive screening of crystallization conditions for EPCR. We obtained crystals in varied conditions and determined the structure of EPCR in three different space groups, which enabled a wider sampling of the EPCR-lipid complexes. In all cases, and in agreement with the mass spectrometry results and previous crystallographic data^23,44,45^, we observe a predominant phospholipid scaffold in all EPCR structures, regardless of the space group. Yet, the precise identity of the outermost fraction bound to the phosphate is ambiguous, which supports the presence of a heterogeneous phospholipid population bound to EPCR.

Analysis of the ADPs revealed a tendency to higher mobility in the lipid region proximal to the groove opening, i.e., the polar head group. This was expected as the buried fatty acids are embedded inside a hydrophobic core, and likely tightly gripped to the receptor. The polar region is in a more solvent-exposed and less locked scenario, hence, the higher static disorder.

As for the morphology of the cavity, it resembles in all cases that of the MHC class I-like CD1 family of receptors, with two major A’ and F’ pockets that provide room for binding of fatty acids. Additional space is found beyond these pockets in some, but not all, EPCR structures. However, these smaller cavities are surrounded by polar residues and therefore their chemistry is not optimal for accommodation of hydrophobic acyl chains. Further, the lack of electron density signal for side chains in the cavity suggest structural plasticity or disorder, *i.e.,* the morphology of EPCR’s cavity is not absolutely rigid. Whether these additional pockets could play a role in EPCR’s functions requires further investigation.

In conclusion, we provide a comprehensive analysis of the lipids found in EPCR. Although with different abundances, EPCR hosts lipids of varied classes. Given the impact that well-defined lipids have on EPCR, including relevant roles in coagulation disorders and autoimmune disease, these findings accentuate the need to investigate the lipid-dependent properties of EPCR and their potential links with disease.

## Materials and methods

### Recombinant EF3 and 3C expression and purification

EF3 cDNA (Invitrogen) was N-terminal fused to MBP cDNA previously isolated from pMBP-MS2 plasmid (Josep Vilardell, Addgene plasmid # 65,104) by PCR. The fusion protein MBP/EF3 and 3C protease cDNA (Invitrogen) was PCR amplified to introduce a N-terminal NcoI restriction site and C-terminal XhoI restriction site. The resulting PCR amplified DNA was digested using FastDigest NcoI and XhoI (Thermo Fisher Scientific) restriction enzymes and cloned in frame with a C-terminal 6 × Histidine tag sequence containing pET-28b( +) transfer vector (kindly provided by Dr. Lasa, Navarrabiomed) using Optizyme™ T4 DNA Ligase (Thermo Fisher Scientific). Competent DH5α *E. coli* cells (Invitrogen) were transformed with the modified transfect vectors and positive clones were grown under kanamycin (50 µg/mL, Panreac AppliChem) selection. Plasmid DNA was extracted using the GeneJET Plasmid Miniprep Kit (Thermo Fisher Scientific) following manufacturer’s instructions. All sequences were validated by Sanger sequencing before transformation of BL21 competent cells (Agilent). A culture of transformed BL21 cells was initiated at 37 °C and 225 rpm and when the optical density at 600 nm reached 0.8, expression of the protein was induced 1 mM isopropyl β-d-1-thiogalactopyranoside (Thermo Fisher Scientific) during 4 h for 3C protease and 1 h for MBP-EF3 at 37 °C and 225 rpm. Cells were centrifuged at 4500 g during 15 min at 4 °C. Pellet was resuspended with lysis buffer containing 50 mM Tris, 150 mM NaCl, pH 7.4, PMSF (Phenylmethanesulfonyl fluoride) 1 mM (Thermo Fisher Scientific), Lysozyme 0.25 mg/mL (Sigma), DTT 1 mM (Thermo Fisher Scientific) and one platelet of EDTA free protease inhibitor cocktail (Sigma) and magnetic stirred for 20 min on ice. The mixture was sonicated at 40% amplitude for 5 min (30 s burst/rest intervals). The sonicated sample was centrifuged at 10,000 g for 20 min. Supernatants were collected and 1 mM DTT 1 mM, 20 mM imidazole (Sigma) were added to 3C protease supernatant and 1 mM EDTA (Avantor) was added to MBP-EF3 supernatant. After filtering the sample through a 0.45 µm membrane (Millipore), the 3C protease sample was loaded onto a Nickel Trap column (ABT) and eluted with 200 mM imidazole. The eluted fraction was further purified by size exclusion chromatography in a Superdex 200 10/300 GL column (Cytiva) equilibrated with 50 mM Tris, 150 mM NaCl, 10 mM EDTA, 1 mM DTT, pH 8.0. MBP-EF3 sample was loaded into an MBPTrap column (Cytiva) and eluted with 10 mM maltose (Alfa Aesar). The eluted sample was desalted to 20 mM Hepes pH 7.4, 150 mM NaCl with a 10,000 kDa cutoff centrifugal filter unit (Thermo Fisher Scientific). Purified proteins were frozen with liquid nitrogen and stored at − 80 °C until used for enzymatic digestion.

### Cloning and generation of recombinant baculovirus

We amplified the gene encoding the extracellular region of EPCR from human cDNA (Genscript) via PCR, and introduced a 6xHis N-terminal tag followed by a human rhinovirus 3C site. The resulting DNA was digested using FastDigest BamHI and NotI (Thermo Fisher Scientific) restriction enzymes and cloned in frame with a N-terminal GP64 secretion signal sequence containing pAcGP67A transfer vector using Optizyme™ T4 DNA Ligase (Thermo Fisher Scientific). Competent DH5α *E. coli* cells (Invitrogen) were transformed with the modified transfer vectors and positive clones were grown under ampicillin selection. Plasmid DNA was extracted using the GeneJET Plasmid Miniprep Kit (Thermo Fisher Scientific) following manufacturer’s instructions. All sequences were validated by Sanger sequencing before transduction into Sf9 insect cells (Gibco™) together with BestBac 2.0 Δ v-cath/chiA linearized baculovirus DNA and Expres2 TR Transfection Reagent (Expression Systems) to produce the final recombinant baculovirus.

### Recombinant expression of EPCR

Sf9 cell cultures in Insect-XPRESS medium (Lonza) supplemented with Penicillin Streptomycin (Fisher) were inoculated with the corresponding baculovirus carrying the gene of interest and left in agitation for 72 h at 28 °C. The culture media was freed of cells and the clarified supernatant concentrated prior to loading onto a Nickel NTA Agarose prepacked resin (ABT) for protein purification using buffers recommended by the manufacturer. Eluted fractions containing protein were pooled and loaded into a HiPrep 26/10 Desalting column (Cytiva) equilibrated with 20 mM TRIS pH 8.0 buffer for desalting. Ionic exchange chromatography was carried out in a HiTrap CaptoQ ImpRes column (Cytiva) to remove remaining impurities. EPCR was further purified by size exclusion chromatography using a Superdex 200 10/300 GL column (Cytiva). All purification steps were performed in an AKTA Pure platform (Cytiva). The purified protein was concentrated using Amicon^®^ Ultra-4 mL devices (Millipore Merck) with a molecular weight cutoff of 10 kDa. For crystallization purposes, the EPCR sample was further processed to remove the N-terminal 6xHisTag using a 50:1 (w/w) ratio with 3C enzyme overnight at 4 °C. The digested product was loaded onto a 1-mL nickel GraviTrap column (Cytiva) and collected in the flow-through. EPCR was then treated with endoglycosidase F3 (EF3) for reducing the extent of branched glycosylation. Digestion proceeded for 18 h at RT using a 1:10 (w/w) enzyme:EPCR ratio. EPCR was isolated from the mixture through an ionic exchange in a MonoQR 5/50 column and applying a 20-mL gradient from 0 to 0.3 M NaCl. The double-digested product was subjected to a final concentration step in a Nanosep 10 kDa cutoff device and used for crystallization trials.

### Crystallization and collection of diffraction data

The EPCR sample at 5 mg/mL was screened against a broad (> 700) array of crystallization cocktails by the vapour diffusion sitting drop method. Initial hits were optimized in order to obtain larger crystals. These were soaked in mother liquor containing 20% glycerol or 20% ethylene glycol and cryo-cooled in liquid nitrogen. Crystal diffraction and data collection was performed at the BL13-XALOC Beamline in the ALBA Synchrotron and the Swiss Light Source X06DA – PXIII beamline at 100 K. The best crystals, which allowed collection of full datasets to 1.95 Å, grew in 2.8 M sodium acetate pH 8.0, 0.1 M TRIS pH 7.0 (P2_1_2_1_2_1_ space group), 2.8 M sodium acetate pH 8.0 (C222_1_ space group) and 2.0 M sodium formate, 0.1 M sodium acetate pH 4.6 (P3_1_21 space group).

### Structure determination and refinement

Data reduction was performed with XDS^46^, and scaled with Aimless^47^ in the CCP4 Suite^48^, A 5% of reflections were excluded for validation purposes during the refinement process. Structures were solved by molecular replacement via Phaser^49^ using as template previously deposited coordinates for EPCR (PDB accession number 1LQV), and omitting all ligand, waters and carbohydrate coordinates.

Refinement was carried out with Phenix.refine^50^ and Refmac5^51^. Refinement strategies included XYZ and individual B-factors, optimization of X-ray/stereochemistry weight and X-ray/ADP weight and the use of non-crystallographic symmetry (NCS) for those datasets with more than one molecule per asymmetric unit. Initial steps were performed using rigid body refinement. Final model was generated through alternate manual building in Coot^52^ and refinement with additional translation-libration-screw step. Lipid ligands and water molecules were added guided by Fo-Fc positive density maps in Coot.

### Mass spectrometry analyses

#### Chemicals

The organic solvents used were MS grade. Methanol (MeOH), acetonitrile and isopropanol were from Fisher Chemical (Pennsylvania, United States). Ammonia (28%, GPR rectapuR) and glacial acetic acid (AnalaR NORMAPUR) were from VWR Chemicals BDH (Pennsylvania, United States). Methyl *tert*-butyl ether (MTBE) and ammonium fluoride (ACS reagent, ≥ 98%) were from Sigma-Aldrich (Sigma‐Aldrich Chemie GmbH, Steinheim, Germany). Reference mass solutions for LC–MS were from Agilent Technologies. Ultrapure water (Milli-Qplus185 system Millipore, Billerica, MA, USA) was used in the preparation of mobile phase A.

#### Sample preparation

The EPCR lipid moiety was extracted following standard isolation procedures with organic solvents^53,54^. Organic fractions were dried under nitrogen stream. Once dried, samples were resuspended in 100 μL MTBE:MeOH (1:1) and analyzed in the LC–MS system.

#### Analytical setup

The analysis of the samples was accomplished using an UHPLC system (1290 Infinity II system, Agilent Technologies, Waldbronn, Germany) coupled to a 6560 Ion Moblity LC/QTOF (Agilent Technologies) with an ESI ion source. The sample injection volume was set up to 1 μL for the analyses in positive ionization mode and 2 μL for the negative ionization mode. The separation was achieved using an InfinityLab Poroshell 120 EC-C18 3.0 × 100 mm, 2.7 µm (Agilent Technologies)) reverse phase column, at 50 °C. The chromatography was based on a mobile phase A (10 mM ammonium acetate, 0.2 mM ammonium fluoride in 9:1 water/methanol) and a mobile phase B (10 mM ammonium acetate, 0.2 mM ammonium fluoride in 2:3:5 acetonitrile/methanol/isopropanol) pumped at 0.6 mL/min. The chromatography gradient began with 70% of phase B, decreasing to 86% B in minute 3.5 and maintained for 6.5 min until minute 10. The gradient then increased to 100% B in minute 11 and was maintained until minute 17. Finally, it decreased to 70% B in minute 17.10 and maintained till minute 19. Separate analyses were performed in order to collect the data in positive and negative ionization modes. These analyses were done in full scan mode with a mass range of 40 to 1800 m/z for both modes. The capillary voltage was set to 3500 V for both modes. The drying gas flow rate was 10 L/min at a temperature of 200 °C, the gas nebulizer was set at 50 psi, and the fragmentor voltage at 400 V. For the IM mobility analyses, data was recorded at 1 frame/s, having 16 frames/frame and using 60 ms as the maximum drift time. The trap fill time was 20,000 µs and the trap release time was 150 µs. The following values were used for positive ionization mode, using the corresponding negative values for negative ionization mode: The high-pressure funnel delta was 150 V, the trap funnel delta was 180 V and the trap funnel exit was 10 V. The drift tube entrance was 1700 V and the exit was 250 V. Having a drift tube of, approximately, 78 cm, the field stablished was, approximately, 18.59 V/cm. The determination of the collision cross section (CCS) values was made by a CCS single-field calibration, by using the masses included in the Agilent Tuning Mix solution. Three reference masses were used per ionization mode in order to provide a constant mass correction: m/z 121.0509 and m/z 140.1437 and m/z 922.0098 for the positive ionization mode, and m/z 112.9856 and m/z 966.0007 and m/z 1033.9881 for the negative mode.

## Supplementary Information

Supplementary Information.

## Data Availability

Atomic coordinates and structure factors for EPCR in space groups P3_1_21, C222_1_, P2_1_2_1_2_1_ and for Tween20-treated EPCR have been deposited in the Protein Data Bank under the accession codes 7OKS, 7OKT, 7OKU and 7OKV, respectively.

## References

[1] Fukudome K, Esmon CT (1994). Identification, cloning, and regulation of a novel endothelial cell protein C/activated protein C receptor. J. Biol. Chem..

[2] Feistritzer C, Sturn DH, Kaneider NC, Djanani A, Wiedermann CJ (2003). Endothelial protein C receptor-dependent inhibition of human eosinophil chemotaxis by protein C. J. Allergy Clin. Immunol..

[3] Sturn DH (2003). Expression and function of the endothelial protein C receptor in human neutrophils. Blood.

[4] Galligan L (2001). Characterization of protein C receptor expression in monocytes. Br. J. Haematol..

[5] Xue M, Campbell D, Sambrook PN, Fukudome K, Jackson CJ (2005). Endothelial protein C receptor and protease-activated receptor-1 mediate induction of a wound-healing phenotype in human keratinocytes by activated protein C. J. Invest. Dermatol..

[6] Gorbacheva L (2009). Endothelial protein C receptor is expressed in rat cortical and hippocampal neurons and is necessary for protective effect of activated protein C at glutamate excitotoxicity. J. Neurochem..

[7] Balazs AB, Fabian AJ, Esmon CT, Mulligan RC (2006). Endothelial protein C receptor (CD201) explicitly identifies hematopoietic stem cells in murine bone marrow. Blood.

[8] Iwasaki H, Arai F, Kubota Y, Dahl M, Suda T (2010). Endothelial protein C receptor-expressing hematopoietic stem cells reside in the perisinusoidal niche in fetal liver. Blood.

[9] Wang J, Yang L, Rezaie AR, Li J (2011). Activated protein C protects against myocardial ischemic/reperfusion injury through AMP-activated protein kinase signaling. J. Thromb. Haemost..

[10] Bretschneider E (2007). Human vascular smooth muscle cells express functionally active endothelial cell protein C receptor. Circ. Res..

[11] Crawley JT, Gu JM, Ferrell G, Esmon CT (2002). Distribution of endothelial cell protein C/activated protein C receptor (EPCR) during mouse embryo development. Thromb. Haemost..

[12] Cheng T (2003). Activated protein C blocks p53-mediated apoptosis in ischemic human brain endothelium and is neuroprotective. Nat. Med..

[13] Joyce DE, Gelbert L, Ciaccia A, DeHoff B, Grinnell BW (2001). Gene expression profile of antithrombotic protein c defines new mechanisms modulating inflammation and apoptosis. J. Biol. Chem..

[14] Mosnier LO, Griffin JH (2003). Inhibition of staurosporine-induced apoptosis of endothelial cells by activated protein C requires protease-activated receptor-1 and endothelial cell protein C receptor. Biochem. J..

[15] Mosnier LO, Zlokovic BV, Griffin JH (2007). The cytoprotective protein C pathway. Blood.

[16] Riewald M, Petrovan RJ, Donner A, Mueller BM, Ruf W (2002). Activation of endothelial cell protease activated receptor 1 by the protein C pathway. Science (80-.).

[17] Turner L (2013). Severe malaria is associated with parasite binding to endothelial protein C receptor. Nature.

[18] Willcox CR (2012). Cytomegalovirus and tumor stress surveillance by binding of a human gammadelta T cell antigen receptor to endothelial protein C receptor. Nat. Immunol..

[19] Mantri CK, St John AL (2018). Immune synapses between mast cells and gammadelta T cells limit viral infection. J. Clin. Invest..

[20] Hurtado V (2004). Autoantibodies against EPCR are found in antiphospholipid syndrome and are a risk factor for fetal death. Blood.

[21] Müller-Calleja N (2021). Lipid presentation by the protein C receptor links coagulation with autoimmunity. Science.

[22] Mutoh T (2020). Identification of two major autoantigens negatively regulating endothelial activation in Takayasu arteritis. Nat. Commun..

[23] Oganesyan V (2002). The crystal structure of the endothelial protein C receptor and a bound phospholipid. J. Biol. Chem..

[24] López-Sagaseta J (2012). sPLA2-V inhibits EPCR anticoagulant and antiapoptotic properties by accommodating lysophosphatidylcholine or PAF in the hydrophobic groove. Blood.

[25] Bouwens EA, Mosnier LO (2012). EPCR encryption induces cellular APC resistance. Blood.

[26] Zajonc DM (2016). The CD1 family: serving lipid antigens to T cells since the Mesozoic era. Immunogenetics.

[27] Adams EJ, Lopez-Sagaseta J (2011). The immutable recognition of CD1d. Immunity.

[28] Adams EJ, Luoma AM (2013). The adaptable major histocompatibility complex (MHC) fold: structure and function of nonclassical and MHC class I-like molecules. Annu. Rev. Immunol..

[29] Nayak RC (2009). Endothelial cell protein C receptor cellular localization and trafficking: potential functional implications. Blood.

[30] Moody DB, Porcelli SA (2003). Intracellular pathways of CD1 antigen presentation. Nat. Rev. Immunol..

[31] Stow SM (2017). An Interlaboratory Evaluation of Drift Tube Ion Mobility-Mass Spectrometry Collision Cross Section Measurements. Anal. Chem..

[32] Scharf L (2010). The 2.5 Å structure of CD1c in complex with a mycobacterial lipid reveals an open groove ideally suited for diverse antigen presentation. Immunity.

[33] Spronk HMH, ten Cate H, van der Meijden PEJ (2014). Differential roles of tissue factor and phosphatidylserine in activation of coagulation. Thromb. Res..

[34] Ross, T. S. *Cellular and Molecular Mechanisms of Inflammation*. 153–174 (1992).

[35] Mallevaey T (2011). A molecular basis for NKT cell recognition of CD1d-self-antigen. Immunity.

[36] López-Sagaseta J (2007). Binding of factor VIIa to the endothelial cell protein C receptor reduces its coagulant activity. J. of Thromb. Haemost..

[37] Preston RJ (2006). Multifunctional specificity of the protein C/activated protein C Gla domain. J Biol Chem.

[38] Ghosh S, Pendurthi UR, Steinoe A, Esmon CT, Rao LV (2007). Endothelial cell protein C receptor acts as a cellular receptor for factor VIIa on endothelium. J. Biol. Chem..

[39] Lau CK (2015). Structural conservation despite huge sequence diversity allows EPCR binding by the PfEMP1 family implicated in severe childhood malaria. Cell Host Microb..

[40] Borg NA (2007). CD1d-lipid-antigen recognition by the semi-invariant NKT T-cell receptor. Nature.

[41] López-Sagaseta, J., Kung, J. E., Savage, P. B., Gumperz, J. & Adams, E. J. The Molecular Basis for Recognition of CD1d/α-Galactosylceramide by a Human Non-Vα24 T Cell Receptor. **10**, (2012). 10.1371/journal.pbio.1001412 PMC3479090 23109910

[42] Cox D (2009). Determination of cellular lipids bound to human CD1d molecules. PLoS ONE.

[43] Marheineke K, Grünewald S, Christie W, Reiländer H (1998). Lipid composition of Spodoptera frugiperda (Sf9) and Trichoplusia ni (Tn) insect cells used for baculovirus infection. FEBS Lett..

[44] Sampath S (2015). Plasmodium falciparum adhesion domains linked to severe malaria differ in blockade of endothelial protein C receptor. Cell. Microbiol..

[45] Vadivel K (2013). Structural and functional studies of γ-carboxyglutamic acid domains of factor VIIa and activated Protein C: role of magnesium at physiological calcium. J. Mol. Biol..

[46] Kabsch W (2010). XDS. Acta Crystallogr. D Biol. Crystallogr..

[47] Evans PR, Murshudov GN (2013). How good are my data and what is the resolution?. Acta Crystallogr. D. Biol. Crystallogr..

[48] The CCP4 suite: programs for protein crystallography. *Acta Crystallogr. D Biol. Crystallogr.* **50**, 760–763 (1994). 10.1107/S0907444994003112 15299374

[49] McCoy AJ (2007). Phaser crystallographic software. J. Appl. Crystallogr..

[50] Adams PD (2010). PHENIX: A comprehensive Python-based system for macromolecular structure solution. Acta Crystallogr. D Biol. Crystallogr..

[51] Kovalevskiy O, Nicholls RA, Murshudov GN (2016). Automated refinement of macromolecular structures at low resolution using prior information. Acta Crystallogr. Sect. D Struct. Biol..

[52] Emsley P, Lohkamp B, Scott WG, Cowtan K (2010). Features and development of Coot. Acta Crystallogr. D Biol. Crystallogr..

[53] Cham BE, Knowles BR (1976). A solvent system for delipidation of plasma or serum without protein precipitation. J. Lipid Res..

[54] Bligh EG, Dyer WJ (1959). A rapid method of total lipid extraction and purification. Can. J. Biochem. Physiol..

[55] Volkamer A, Griewel A, Grombacher T, Rarey M (2010). Analyzing the topology of active sites: on the prediction of pockets and subpockets. J. Chem. Inf. Model..

