# Supplementary material for: Identification of a broad lipid repertoire associated to the endothelial cell protein C receptor (EPCR)
Source: Sci Rep. 2022 Sep 6;12:15127. doi: 10.1038/s41598-022-18844-y (PMC9448719; doi:10.1038/s41598-022-18844-y)
Supplement: Supplementary file 1 — Supplementary Information. [file 41598_2022_18844_MOESM1_ESM.pdf]

Supplementary Materials

Identification of a broad lipid repertoire associated to the endothelial cell protein C receptor (EPCR)

Elena Erausquin[1,2,3†], María Morán-Garrido[4†], Jorge Saiz[4], Coral Barbas[4], Gilda Dichiara-Rodríguez[1,2,3], Alejandro Urdiciain[1,2,3] and Jacinto López-Sagaseta[1,2,3 *]

[1] Unit of Protein Crystallography and Structural Immunology, Navarrabiomed, 31008, Navarra, Spain.
[2] Public University of Navarra (UPNA), Pamplona, 31008, Navarra, Spain.
[3] Navarra University Hospital, Pamplona, 31008, Navarra, Spain.
[4] Centro de Metabolómica y Bioanálisis (CEMBIO), Facultad de Farmacia, Universidad San Pablo-CEU, CEU Universities, Urbanización Montepríncipe, 28660, Boadilla del Monte, Spain.

[†] Contributed equally.

[*] To whom correspondence should be addressed: jacinto.lopez.sagaseta@navarra.es

Supplementary Figures 1 to 4.

Supplementary Table 1.

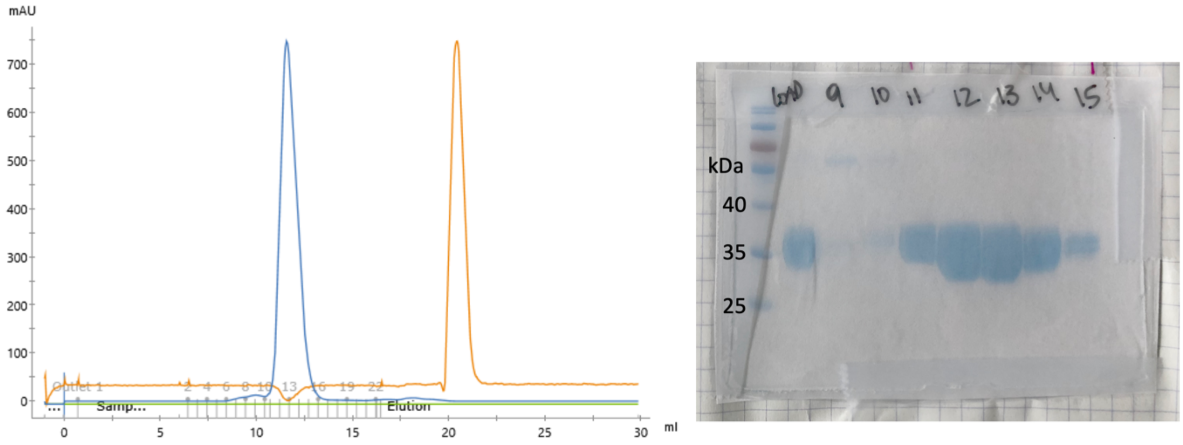

Supplementary Fig. 1. Purification of EPCR tagged with Twin-Strep. Left, size exclusion chromatography (S75 10/300 Increase, Cytiva) of the biotin-eluted fraction recovered from a Strep-Tactin™XT Superflow™ resin. Right, SDS-PAGE of the initial sample and fractions 9 to 15.

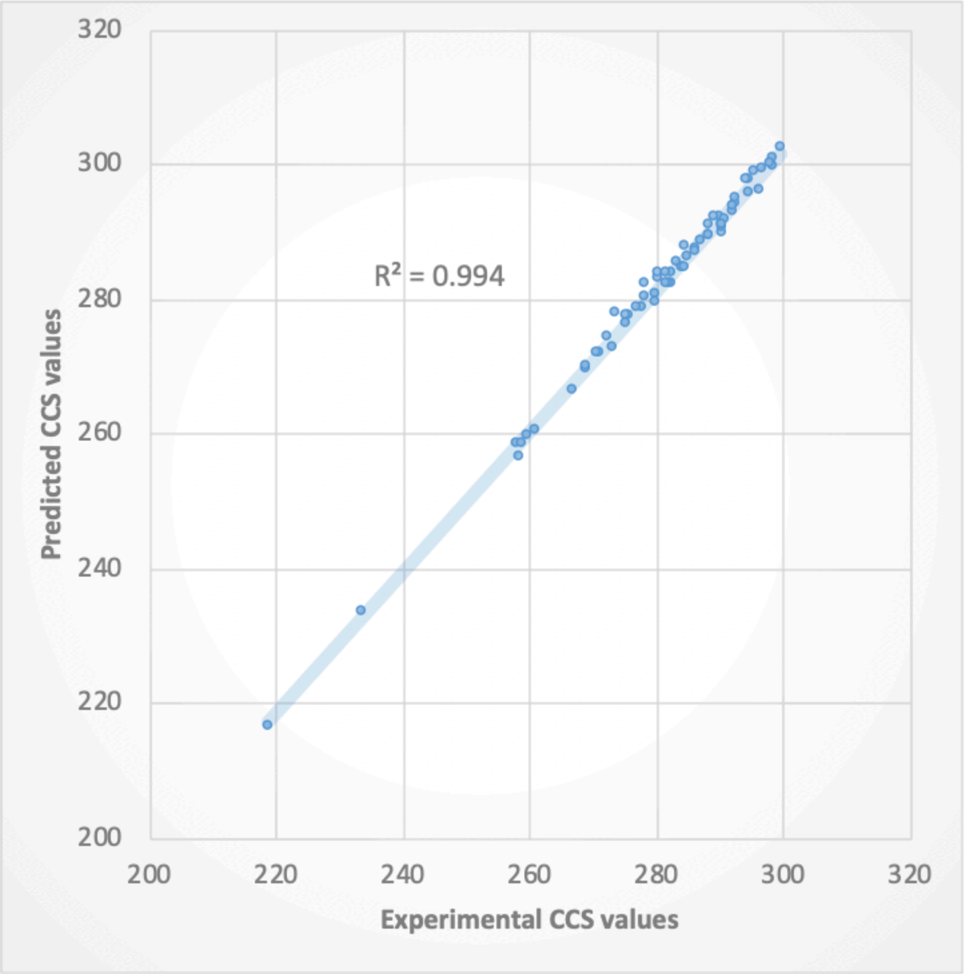

Supplementary Fig. 2. Correlation between predicted and observed CCS values. Fitting of the experimental CCS values for lipids obtained in this work with the CCS values provided by CCSBase.

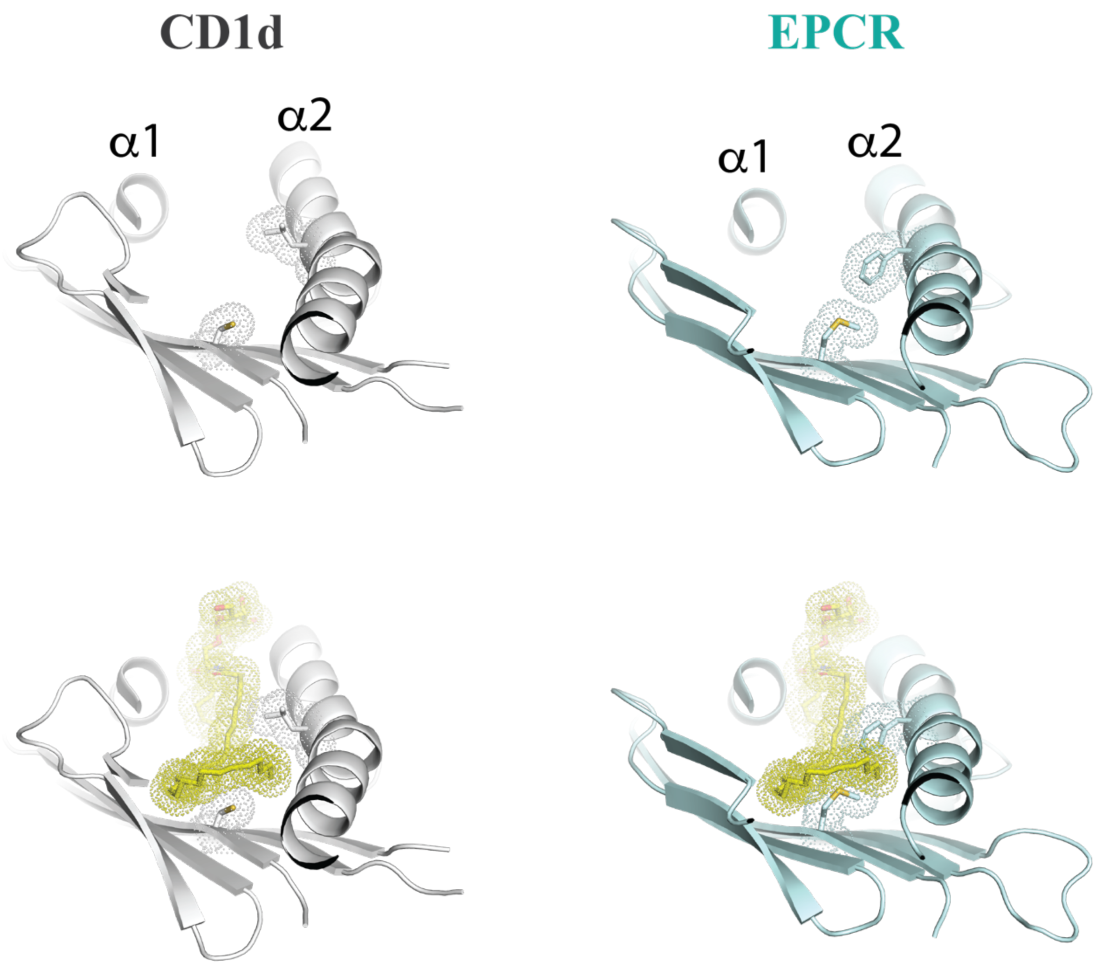

Supplementary Fig. 3. Restriction of lipid tail lengh by EPCR residues in the A' pocket. Left, structure of CD1d with bound alpha-galacatosylceramide (PDB 4EN3). CD1d in shown in gray color. Cys13 and Leu161 side chains are indicated and highlighted with sticks and dots. CD1d favors lipid long acyl chains plasticity to curve and fit within the A' pocket of CD1d. The figure on the left lower panel shows the long acyl chain of alpha-galactosylceramide, bound to CD1d, as yellow sticks and dots surrounded by Cys13 and Leu161. On the right, the equivalent structure of EPCR aligned to CD1d-alpha-galactosylceramide suggests structural restriction for long acyl chains by the bulkier Met13 and Phe164 side chains.

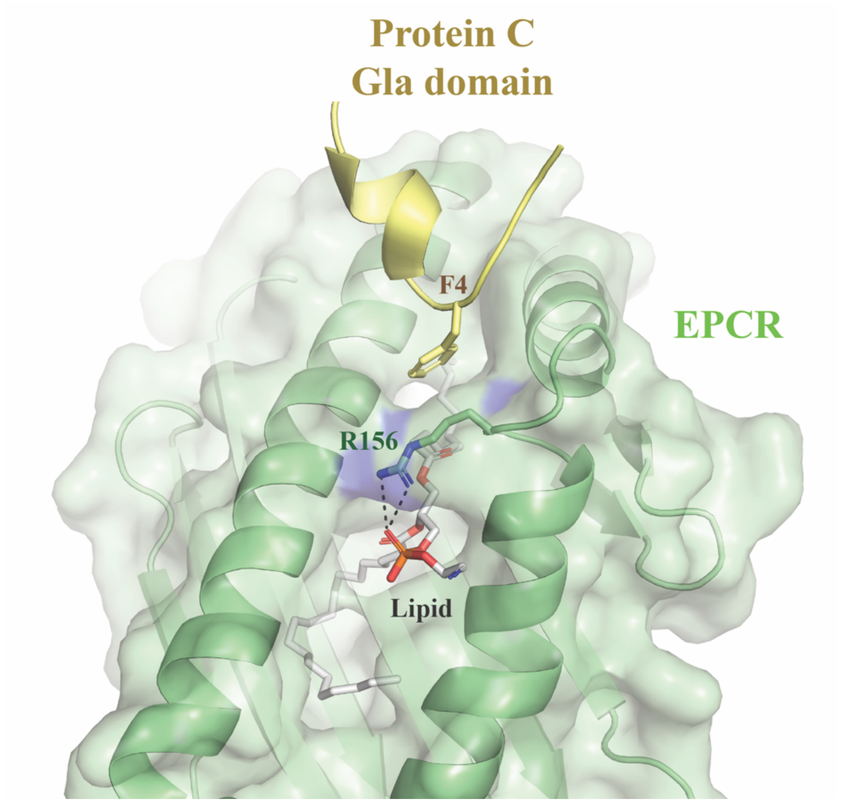

Supplementary Fig. 4. Lipid-mediated restriction for docking of the protein C Gla domain. The image shows the molecular structures of EPCR (in green color) and the protein C Gla domain (in pale yellow color). The EPCR Arg156, Gla Phe4 and the phosphatidylethanolamine lipid are highlighted with sticks. The interactions between Arg156 and the phosphate group in the lipid are displayed as yellow dashed lines. The surface representation of EPCR allows visualization of the pocket enabled by Arg156 conformation, which allows insertion of the protein C Gla Phe4 protein:protein docking. Figure created using the coordinates of the protein C Gla domain-EPCR complex solved by Oganesyan *et al* (doi: 10.1074/jbc.C200163200; PDB 1LQV).

Compound name	Formula	Mass	Polarity	RT (min)	CCS (% error)
Cer(d14:1/22:0)	$C_{36}H_{71}NO_3$	565.5434	NEG	11.7	260.7 (0.00)
DG 16:1_18:1	$C_{37}H_{68}O_5$	592.5067	POS	11.5	258.7 (-0.08)
LPC(0:0/18:1)	$C_{26}H_{52}NO_7P$	521.3481	POS	2.7	
LPC(18:1/0:0)	$C_{26}H_{52}NO_7P$	521.3481	POS	2.9	233.7 (0.13)
LPE(0:0/18:1)	$C_{23}H_{46}NO_7P$	479.3012	POS/NEG	2.8	
LPE(18:1/0:0)	$C_{23}H_{46}NO_7P$	479.3012	POS/NEG	3.0	216.6 (-1.01)
PC(16:0/16:1)	$C_{40}H_{78}NO_8P$	731.5465	POS/NEG	7.8	282.5 (0.14)
PC(16:0/18:1)	$C_{42}H_{82}NO_8P$	759.5778	POS/NEG	9.8	289.6 (0.42)
PC(16:1/16:1)	$C_{40}H_{76}NO_8P$	729.5309	POS/NEG	6.5	280.7 (0.32)
PC(18:0/16:0)	$C_{42}H_{84}NO_8P$	761.5935	POS/NEG	11.4	291.4 (0.31)
PC(18:0/18:1)	$C_{44}H_{86}NO_8P$	787.6091	POS/NEG	11.7	295.9 (0.51)
PC(18:1/16:1)	$C_{42}H_{80}NO_8P$	757.5622	POS/NEG	8.0	287.4 (0.42)
PC(18:1/18:1)	$C_{44}H_{84}NO_8P$	785.5935	POS/NEG	10.1	294.1 (0.62)
PC(18:1/18:2)	$C_{44}H_{82}NO_8P$	783.5778	POS/NEG	8.7	290.7 (0.14)
PC(18:1/20:1)	$C_{46}H_{88}NO_8P$	813.6248	POS/NEG	11.7	299.6 (0.44)
PC(18:2/18:1)	$C_{44}H_{82}NO_8P$	783.5778	POS/NEG	7.5	292.0 (0.59)
PC(18:3/16:1)	$C_{42}H_{76}NO_8P$	753.5309	POS/NEG	5.8	282.5 (0.21)
PC(18:3/18:1)	$C_{44}H_{80}NO_8P$	781.5622	POS/NEG	6.6	289.3 (0.38)
PC(20:5/18:1)	$C_{46}H_{80}NO_8P$	805.5622	POS/NEG	6.9	291.0 (0.17)
PC(22:6/18:1)	$C_{48}H_{82}NO_8P$	831.5778	POS/NEG	7.6	297.6 (0.98)
PE(16:0/16:1)	$C_{37}H_{72}NO_8P$	689.4996	POS/NEG	8.0	269.6 (0.30)
PE(16:0/18:1)	$C_{39}H_{76}NO_8P$	717.5309	POS/NEG	10.2	276.5 (0.47)
PE(16:1/16:1)	$C_{37}H_{70}NO_8P$	687.4839	POS/NEG	6.6	266.6 (0.00)
PE(18:0/18:1)	$C_{41}H_{80}NO_8P$	745.5622	POS/NEG	11.7	282.2 (0.25)
PE(18:1/16:1)	$C_{39}H_{74}NO_8P$	715.5152	POS/NEG	8.3	272.8 (-0.11)
PE(18:1/18:1)	$C_{41}H_{78}NO_8P$	743.5465	POS/NEG	10.5	279.6 (0.00)
PE(18:2/16:1)	$C_{39}H_{72}NO_8P$	713.4996	POS/NEG	7.1	272.0 (0.33)
PE(18:2/18:1)	$C_{41}H_{76}NO_8P$	741.5309	POS/NEG	8.9	278.7 (0.40)
PE(18:3/16:1)	$C_{39}H_{70}NO_8P$	711.4839	POS/NEG	5.8	269.9 (0.33)
PE(18:3/18:1)	$C_{41}H_{74}NO_8P$	739.5152	POS/NEG	7.0	277.7 (0.76)
PE(20:1/18:1)	$C_{43}H_{82}NO_8P$	771.5778	POS/NEG	11.8	287.0 (0.38)
PI(16:1/16:0)	$C_{41}H_{77}O_{13}P$	808.5102	POS/NEG	6.0	288.5 (0.59)
PI(18:0/16:1)	$C_{43}H_{81}O_{13}P$	836.5415	POS/NEG	7.1	294.8 (0.79)
PI(18:0/18:1)	$C_{45}H_{85}O_{13}P$	864.5728	POS/NEG	8.9	301.1 (1.01)
PI(18:0/20:5)	$C_{47}H_{81}O_{13}P$	884.5415	NEG	6.6	300.3 (0.87)
PI(18:1/18:1)	$C_{45}H_{83}O_{13}P$	862.5571	NEG	7.3	299.2 (0.87)
PS(16:1/18:0)	$C_{40}H_{76}NO_{10}P$	761.5207	POS/NEG	7.3	283.1 (1.07)
PS(18:1/16:1)	$C_{40}H_{74}NO_{10}P$	759.505	POS/NEG	6.5	280.4 (0.86)
SM(d18:1/16:0)	$C_{39}H_{79}N_2O_6P$	702.5676	POS/NEG	7.4	284.6 (0.14)

SM(d18:1/18:0)	$C_{41}H_{83}N_2O_6P$	730.5989	POS/NEG	9.4	291.0 (0.24)
SM(d18:1/20:0)	$C_{43}H_{87}N_2O_6P$	758.6302	POS/NEG	11.6	296.3 (0.00)

Supplementary Table 1. Raw lipid formulas, mass, retention times and CCS values with relative error (%). Lipids found in native EPCR extracts, together with their formulas, neutral mass, ionization polarity in which they were detected, adducts found, their retention times using the working conditions described in Material and Methods section and the experimental CCS values compared with the predicted values (%) from CCSBase, https://ccsbase.net/.
